# Dataset for correlation in γ-RbAg_4_I_5_ between ionic conductivity relaxation and specific heat

**DOI:** 10.1016/j.dib.2019.104404

**Published:** 2019-08-20

**Authors:** H. Correa, Alvaro Garcia Muriel, D. Peña Lara

**Affiliations:** a Laboratorio de Optoelectrónica, Universidad del Quindío, Armenia, Colombia; b Departamento de Física, Universidad de Cartagena, Cartagena, Colombia; c Departamento de Física, Universidad del Valle, Cali, Colombia; d Excellence Center for Novel Materials (CENM), Universidad del Valle, Cali, Colombia

**Keywords:** Electric modulus formalism, *β*-correlation function, ac-calorimetry technique

## Abstract

Using the ac-calorimetry technique and the electric modulus formalism for analysis of ionic conductivity relaxation in crystalline γ-RbAg_4_I_5_, close to the γ to β phase transition at 120 K, the temperature derivative of microscopic interaction energy for a single-mobile ion is proportional to the specific heat. The two different experimental techniques show that cooperative behavior drives the phase transition at 120 K (obey the same mechanism).

**Table:** Specifications Table

Subject area	*Physics*
More specific subject area	*Experimental condensed matter*
Type of data	*Graphs, figures*
How data was acquired	*Specific heat measurements, conductivity-frequency profile, raw data were analyzed by Origen software*
Data format	*Raw, analyzed, fitted*
Experimental factors	Data of conductance are computed using KWW model, Jonscher equation, and K. Ngai model
Experimental features	*Specific heat data were taken by an automatized high-resolution ac calorimeter and conductance data by a commercial impedance bridge.*
Data source location	*City of Cali, Colombia*
Data accessibility	*Data are provided in this current article*

**Table:** 

**Value of the data** • Temperature-dependent data of single ion microscopic energy give the vision to explain the dynamics near the γ-to-β first-order transition in crystalline superionic system RbAg_4_I_5_ at 120 K. • It is well known that for the first-order phase transition takes place, the internal energy necessary to obtain it, corresponds to the thermally provided activation energy. The data shows that only the migration energy contributes to the phase transition in this compound. • The data for measurements of specific heat and conductance can be used to demonstrate that both experimental techniques share the same origin.

## 1 Data

**Fig. 1:**
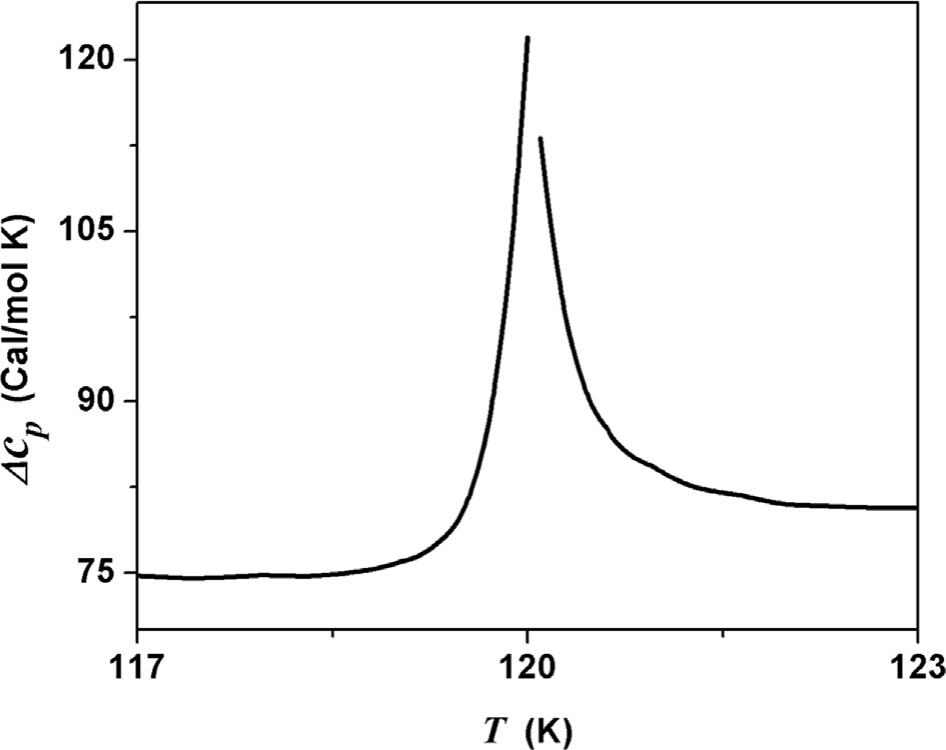
The specific heat at a constant pressure of RbAg_4_I_5_ as a function of temperature. At 120 K, the first-order phase transition occurred.

Fig. 1 shows a discontinuous change of the dc-conductivity with an associated peak in the excess specific heat of RbAg_4_I_5_ where the first-order phase transition occurs at 120 K or phase boundary is between the γ- RbAg4I5 and β- RbAg_4_I_5_ [1], [2], [3]. The value of enthalpy corresponding to the phase transition is provided by the migration energy, which allows us to correlated both thermodynamics and transport concepts.

## 2 Experimental design, materials and methods

Using the solution technique with high purity reagents [4], the RbAg_4_I_5_ crystals at 318 K and dried at 390 K for about 6 hours were grown. For the crystallographic analysis, the crystal samples are a representative specimen.

Using the ac-calorimetry technique [5], [6], the specific heat data of RbAg_4_I_5_ single crystals were continuously obtained. Using dry abrasives crystal slices were thinned to 0.1 mm. By light chopped at 1.5 Hz, the sample was heated. Using a 25 μm type-K thermocouple, the temperature oscillations induced in the sample, inversely proportional to the specific heat, were monitored. The sample was swept slowly through the region of the phase transitions to obtain the specific heat at constant pressure $c_{p}\left(T\right)$ as a function of the temperature ***T***.

Using the two-electrode configuration Ag|RbAg_4_I_5_|Ag with silver paste as electrodes, an electrical measurement was made. By admittance spectroscopy in 20 Hz to 3 MHz frequency range, using a precision LCR meter HP 4284A and at different fixed temperatures between 105 K and 121 K, under a dry nitrogen atmosphere, the electrical characterization was done. The amplitude of the applied ac signal was 10 mV.

**Fig. 2:**
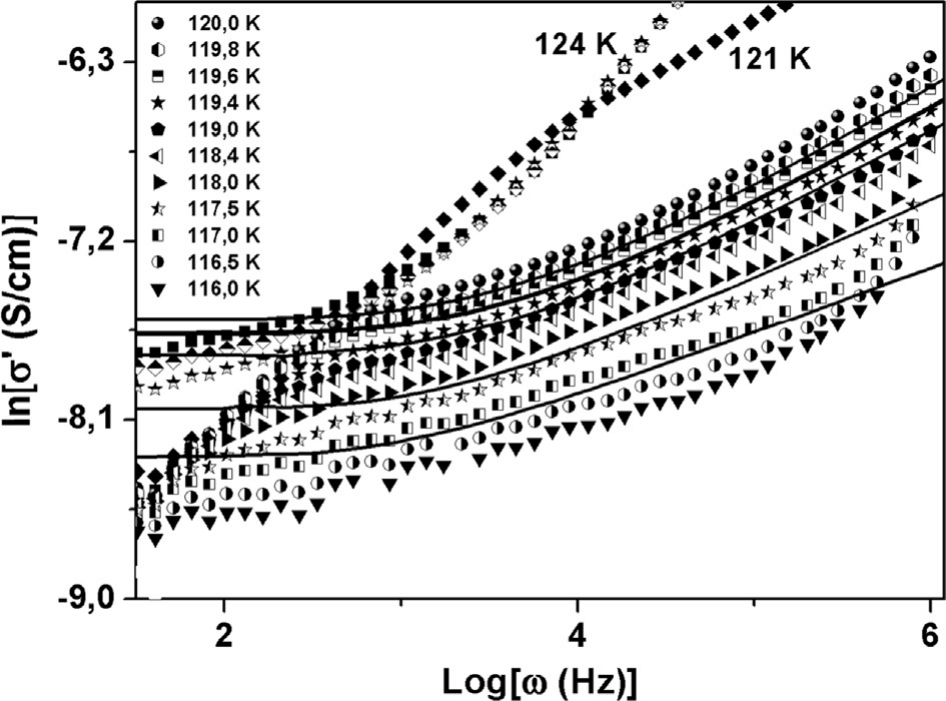
Real part of the electrical conductivity as a function of frequency for (116–124) K temperature range.

In the crossover region, the dependence of the real part of the ac conductivity σ'(T,ω), is described for ionic conducting materials by a power law [7]:

(1) $$\sigma^{'}\left(T,\omega \right)=\sigma_{0}\left(T\right)\left[1+{\left(\frac{\omega }{\omega_{p}\left(T\right)}\right)}^{n}\right]$$

where $\omega_{p}$ is a characteristic relaxation, $\sigma_{0}$ is the dc conductivity, and ***n*** is the power-law exponent related to the degree of correlation among moving ions [8]. The frequency dependence of the real part of the ac conductivity for isotherms in the 116 k to 124 K temperature range is shown in Fig. 2.

**Fig. 3:**
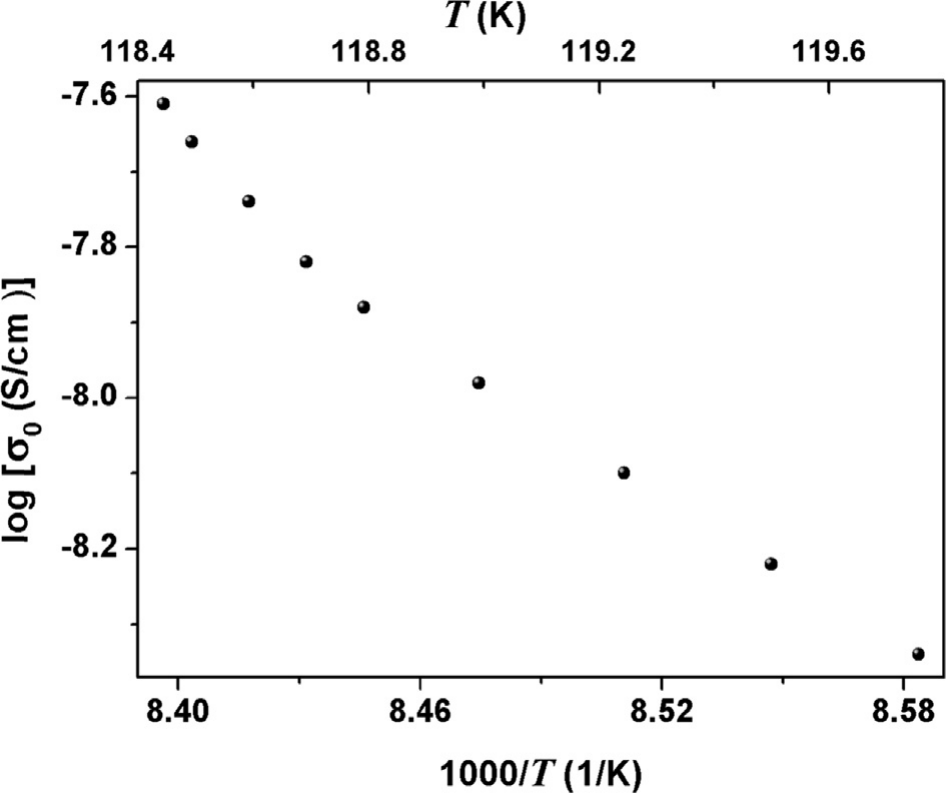
Real part of the electrical conductivity as a function of the inverse of temperature.

The parameters $\omega_{p}\left(T\right)$ and $\sigma_{0}\left(T\right)$ were obtained by fitting the $\sigma^{'}\left(\omega \right)$ data at various isotherms according to

(2) $$M^{*}\left(\omega \right)=\frac{1}{\varepsilon^{*}\left(\omega \right)}=\frac{i\varepsilon_{0}\omega }{\sigma^{*}\left(\omega \right)}$$

and the Arrhenius plot [log($\sigma_{0}$) as a function of 1000/T] is shown in Fig. 3.

Activation energy, $E_{act}=\partial \ln\left(\sigma_{0}\right)/\partial \left(1/T\right)$, is non-Arrhenius in the 118.4 k to 119.8 K temperature range for dc-conductivity data.

**Fig. 4:**
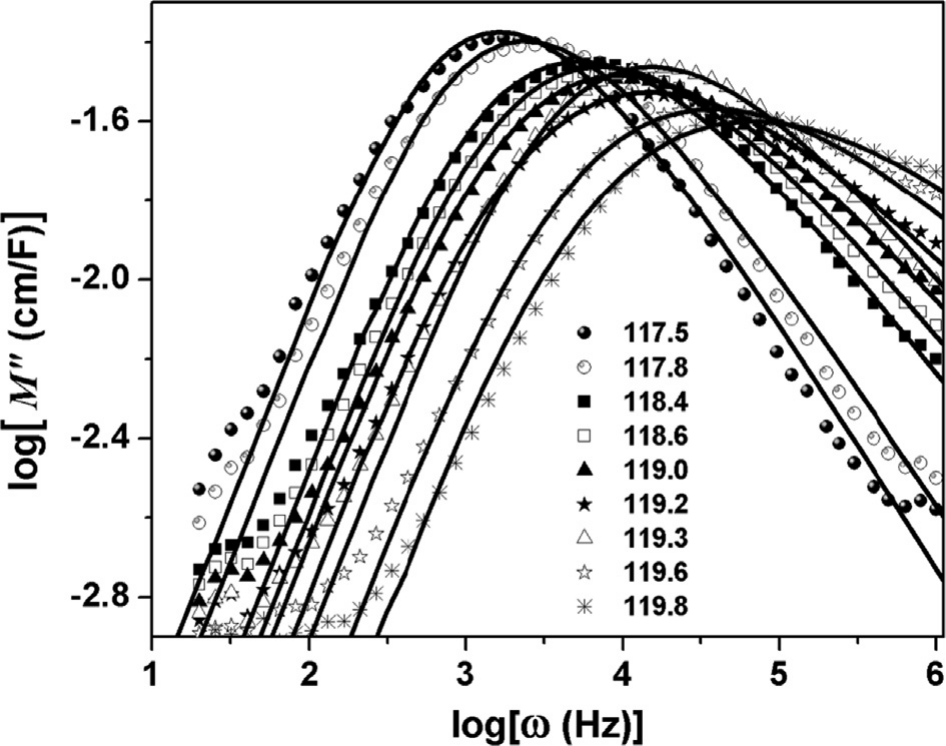
Dependence of frequency of the real part of the electrical modulus for 117.5 and 119.8 K temperature range.

**Fig. 5:**
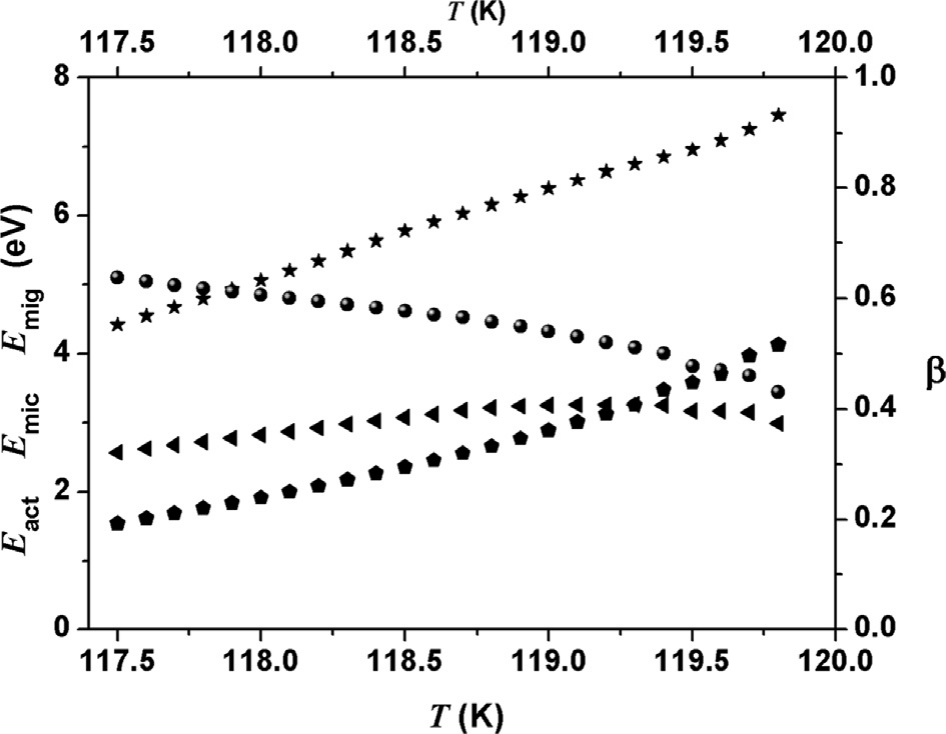
β-correlation function, the activation, microscopic and migration energies as a function of temperature.

Frequency dependence of the imaginary part of the dielectric modulus is shown in Fig. 4 at several temperatures range (117.5 K and 119.8 K):

β-correlation function, the activation, microscopic and migration energies as a function of temperature is shown in Fig. 5 for 117.5 K and 119.8 K temperature range.

**Fig. 6:**
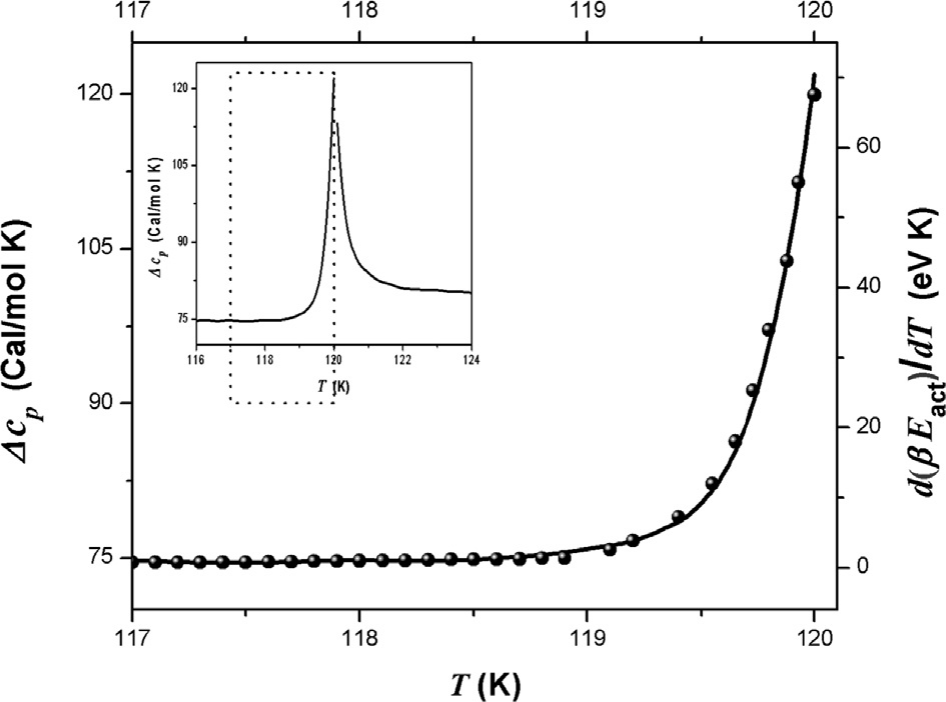
Results of d(β*E*_act_)/*dT* (solid line) and $\Delta c_{p}\left(T\right)$ (filled spheres) as a function of temperature near and below transition region.

Results of d(β*E*_act_)/*dT* and $\Delta c_{p}$ is shown in Fig. 6 the where these quantities exhibit similar behavior with temperature.
